# Optimized medium composition in *Physalis alkekengi* callus culture altered nitric oxide level for inducing antioxidant enzyme activities and secondary metabolites

**DOI:** 10.1038/s41598-024-67191-7

**Published:** 2024-07-16

**Authors:** Halimeh Hassanpour

**Affiliations:** Aerospace Research Institute, Ministry of Science Research and Technology, Tehran, 14665-834 Iran

**Keywords:** Callus induction, Casein hydrolysate, Basal media, *Physalis alkekengi*, Withanolides, Physiology, Plant sciences

## Abstract

*Physalis alkekengi* L. is a valuable medicinal plant from the Solanaceae family and has multiple therapeutic applications. This study aimed to develop an optimized protocol for callogenesis in *P. alkekengi* to obtain friable calluses with high biomass. The effect of different concentrations of picloram, casein hydrolysate (CH), basal media (Murashige and Skoog (MS) and Gamborg (B5)), and static magnetic field (SMF) were investigated on the callus induction and growth, signaling molecules, and enzymatic and non-enzymatic antioxidants. Results showed that CH (200 mgL^−1^) and SMF4 mT for 90 min increased callus induction and fresh weight in *P. alkekengi*, while different concentrations of picloram reduced callogenesis. Hypocotyl explants showed various callogenesis and metabolic responses depending on the basal medium type. The 2B5 medium supplied with CH 200 (mgL^−1^) induced friable and cream calluses with high biomass (0.62 g) compared to the MS medium (control). The maximum activity of superoxide dismutase and catalase activities was identified in the 2B5 medium and peroxidase in the 2MS medium. The highest total phenolic (129.44 µg g^−1^DW) content and phenylalanine-ammonia lyase activity were obtained in the 2MS medium, and total withanolides (49.86 µg g^-1^DW) and DPPH radical scavenging activity were observed in the 2B5 medium. The 2MS medium boosted the hydrogen peroxide and nitric oxide levels, while their contents alleviated in the 2B5 medium, although these parameters were higher than the control. The findings of this study suggest that an effective protocol for successful callogenesis in *P. alkekengi* and the nutrient composition of culture medium by affecting the level of signaling molecules can control the antioxidant defense system and callus growth.

## Introduction

Medicinal plants are valuable sources of pharmaceutical compounds that are effective in the treatment of diseases. These plants generate many important organic chemicals named secondary metabolites^1^. Winter cherry (*Physalis alkekengi* L.) is a medicinal plant that belongs to the Solanaceae family and is widely used in the traditional medicine and food industries. It is distributed in Europe and Asia, including Korea, Japan, and North America, and grows spontaneously at an altitude of 1500 m in the Alborz mountains of Iran. The fruit and calyx of *P. alkekengi* are used to treat excessive phlegm and sore throat, dysuria, eczema, dumb, hepatitis, and bladder disorders in traditional medicine^2,3^. Some research has reported the anti-inflammatory, anti-microbial^4^, antioxidant^5^, anti-diabetic, anti-leishmanial, and anti-asthmatic activities^6^ of *P. alkekengi* extract and also cytotoxic effects against human cancer cell lines^7^ due to the existence of phenolics, flavonoids, alkaloids, fatty acids, steroids, etc. Steroids are the main and bioactive compounds in *P. alkekengi*, and until now, 164 steroids (about 30.65% of total compounds) have been identified, including physalins, withanolides, neophysalins, etc.^2,7^. Moreover, the artificial production of these metabolites is complex and economically unfeasible. On the other hand, *P. alkekengi* reproduction is extremely difficult and needs mountainous weather conditions. So, biotechnological methods can help to enhance biomass and valuable metabolites in this plant.

Plant cell and tissue culture is a possible industrial procedure to manipulate the valuable metabolite pathways and can help to scale up biomass yield for commercial and research applications^8^. Callus is an unorganized tissue containing a mass of un-differentiated parenchyma cells and can be applied as an effective way to manipulate high-valuable secondary metabolites. Designing a protocol for a successful plant tissue culture is a difficult task, and many factors, including plant material, culture conditions, and media ingredients, interfere in this process. Several methods, including optimizing medium compositions, physical and chemical elicitors, and selection of cell lines have been reported to access the optimum growth and secondary metabolite production^9,10^. Also, different basal media and hormonal compositions are used depending on the plant species and nutrients existing in these media^11^. For example, Lekamge et al.^12^ investigated the impact of different macronutrient elements on the in vitro culture of potatoes and the highest seedling growth and shoot length obtained in the medium of 2.5MS (Murashige and Skoog). Hazrati et al.^13^ showed that the highest hazelnut callus induction and growth were acquired in ^1^/_2_ MS medium supplied with 6-furfurylaminopurine (KIN, 0.2 mg L^−1^) and 2, 4-dichlorophenoxyacetic acid (2,4-D, 2 mg L^−1^), and sonication (1 min). In rice, the MS medium supplied with 2, 4-D (2 mg L^−1^), and 0.6% (w/v) casein hydrolysate (CH) was introduced as the best for callogenesis^14^. In *P. alkekengi*, callogenesis was induced in the 1/2MS medium supplied with 2, 4-D (2 mg L^−1^), which accompanied by organogenesis^15^. There were no reports about the specific basal medium for the friable callus culture of *P. alkekengi*, and further investigation is needed.

Static magnetic field (SMF) is a physical factor affecting cell growth and development, metabolic pathways, and antioxidant compounds through the regulation of the amount and/or activity of free radicals^16,17^. The biological impact of magnetic field (MF) can largely depend on the exposure duration and intensity, plant species, growth stage, etc.^16,18^. In *Nigella sativa,* the maximum callus induction and growth were obtained under MF at 400 mT for 30 min^18^. SMF at 4 mT considerably changed cell biomass and antioxidative enzyme activities at the late logarithmic-growth phase of *Matricaria chamomilla* cell suspension^16^. The permeability of cell membranes and nutrient absorptions also changed under MF^19^. There is little research on the factors affecting on callus induction and growth in *P. alkekengi*. So, the purpose of this study was to optimize the basal media to access friable calli with high growth and secondary metabolite production. The preliminary study showed that the MS medium supplied with 6-benzyl aminopurine (BAP, 1.5 mgL^−1^) and 1-naphthaleneacetic acid (NAA, 0.4 mgL^−1^) induced callogenesis and total withanolide content in *P. alkekengi* (data is publishing). In this study, the impact of various concentrations of 4-amino-3,5,6-trichloropicolinic acid (picloram), CH, basal media, and SMF were studied on the callus induction and growth of *P. alkekengi*, and the mechanisms interfering in the growth, including hydrogen peroxide (H_2_O_2_) and nitric oxide (NO) levels, enzymes activities, and secondary metabolites were investigated.

## Results

### Impact of picloram on the callus induction and growth.

In the current study, different concentrations of picloram, a synthetic auxin, were examined in the MS medium to optimize callus induction and growth. As shown in Table 1, picloram decreased callus growth in *P. alkekengi*, and with the rise in the picloram concentration (1 mgL^−1^), callus induction (43%) and growth (48.3%) more decreased compared to the control condition with the callus status of loose and brownish. Adding BAP to the medium supplied with picloram slightly ameliorated the negative impact of picloram on the growth rate. So, the MS medium supplied with BA (1.5 mgL^−1^) and NAA (0.4 mgL^−1^) was selected as an optimum condition for future study.

**Table 1:** Impact of different growth regulators in the callus induction, fresh weight, and callus status of *Physalis alkekengi.*

Treatment	Plant growth regulators (mgL^−1^)			Callus induction (%)	Fresh weight (g)	Callus status
	BAP	NAA	Picloram			
Con	1.5	0.4	–	100 ± 5.7 a	0.29 ± 0.019 a	Friable, compact, and cream
PIC1	–	–	0.25	100 ± 6.2 a	0.20 ± 0.026 b	Loose and cream
PIC2	–	–	0.5	82 ± 2.6 b	0.18 ± 0.019 bc	Loose and brownish
PIC3	–	–	1	57 ± 4.1 a	0.15 ± 0.012 c	Loose and brownish
PIC4	1.5	–	0.25	100 ± 6.7 a	0.22 ± 0.009 b	Loose and cream
PIC5	1.5	–	0.5	66 ± 3.1 c	0.21 ± 0.021 b	Loose and brownish
PIC6	1.5	–	1	52 ± 3.9 d	0.17 ± 0.018 bc	Loose and brownish

Values are means ± SE of five replicates. Different letters indicated significant (p ≤ 0.05) differences. SE standard error.

Con, control; PIC, picloram; BAP, 6-Benzylaminopurine; NAA, 1-naphthaleneacetic acid.

### Impact of CH and SMF on the callus induction and growth

**Figure 1:**
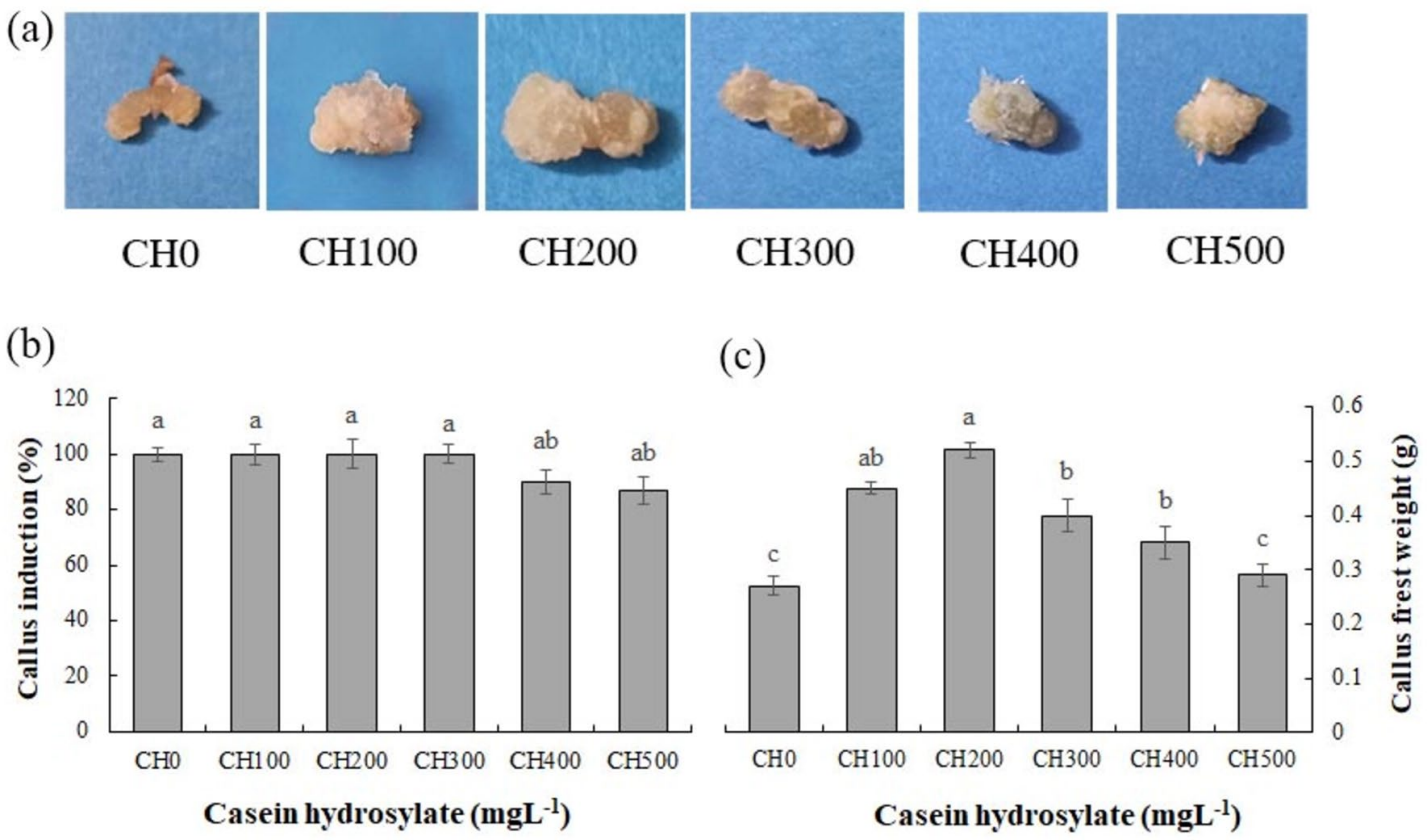
Depiction of *Physalis alkekengi* callus tissues grown under different concentrations of CH, (**a**), the impact of CH on the callus induction (**b**), and callus fresh weight (**c**). Scale bars = 4.5 cm. Values are means ± SE of five replicates. Different letters indicated significant (p ≤ 0.05) differences. SE; standard error, CH; Casein hydrolysate.

As shown in Fig. 1, CH significantly increased callus weight compared to the control. CH at 100 and 200 mgL^−1^ caused a 31.02 and 58.62% rise of callus weight as compared to control, respectively, and the callus was friable and creamy in color. However, the callus weight and induction decreased in the higher concentration of CH (300–500 mgL^−1^). After 3 weeks, their colors trended to get white, and organogenesis (the rooting) initiated in the medium supplied with 400 and 500 mgL^−1^ CH (Fig. 1b and c). According to the results, CH at 200 mgL^−1^ concentration is suggested for application in the medium culture to access the friable calluses in *P. alkekengi*.

**Figure 2:**
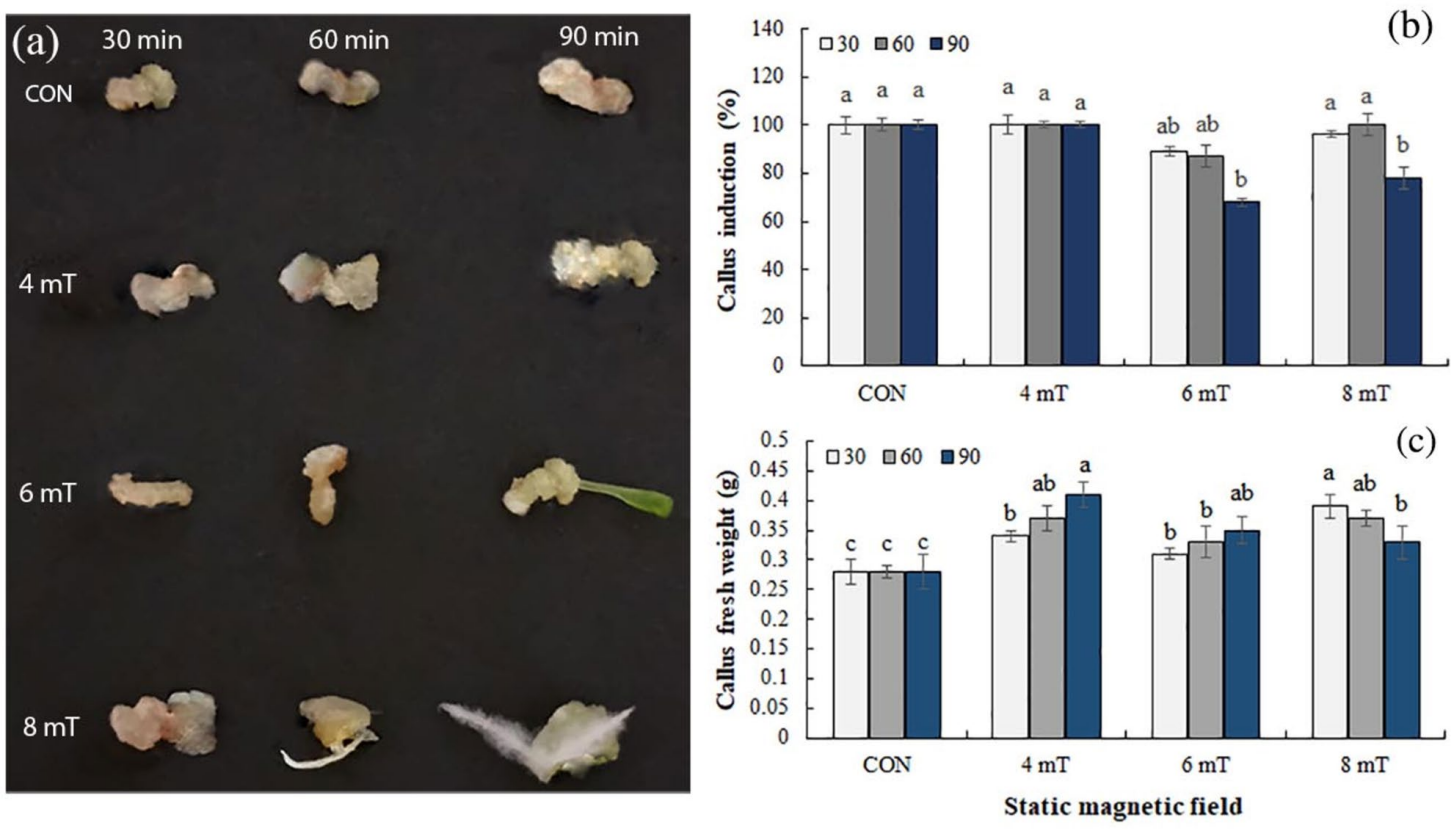
Depiction of *Physalis alkekengi* callus tissues grown under different intensities and durations of SMF (**a**), the impact of SMF on the callus induction (**b**), and callus fresh weight (**c**). Scale bars = 6 cm. Values are means ± SE of five replicates. Different letters indicated significant (p ≤ 0.05) differences. SE; standard error, SMF; Static magnetic field.

SMF application showed various responses of callus induction in *P. alkekengi* (Fig. 2a). Callus growth induced after exposure of hypocotyl explant to SMF at 4 mT, decreased slightly at 6 mT, and then increased at 8 mT. In addition, SMF at 4 mT increased callus fresh weight with the rise of time, and calli had cream in color after 90 min. Treatment of 8 mT for 30 min enhanced callus weight, but with an increase in the SMF duration, callus fresh weight and induction decreased, and organogenesis appeared during callus growth (Fig. 2b and c). The best SMF treatment was identified at 4 mT for 90 min with the maximum callus weight and cream in color.

### Impact of basal media on the callus induction and growth.

**Figure 3:**
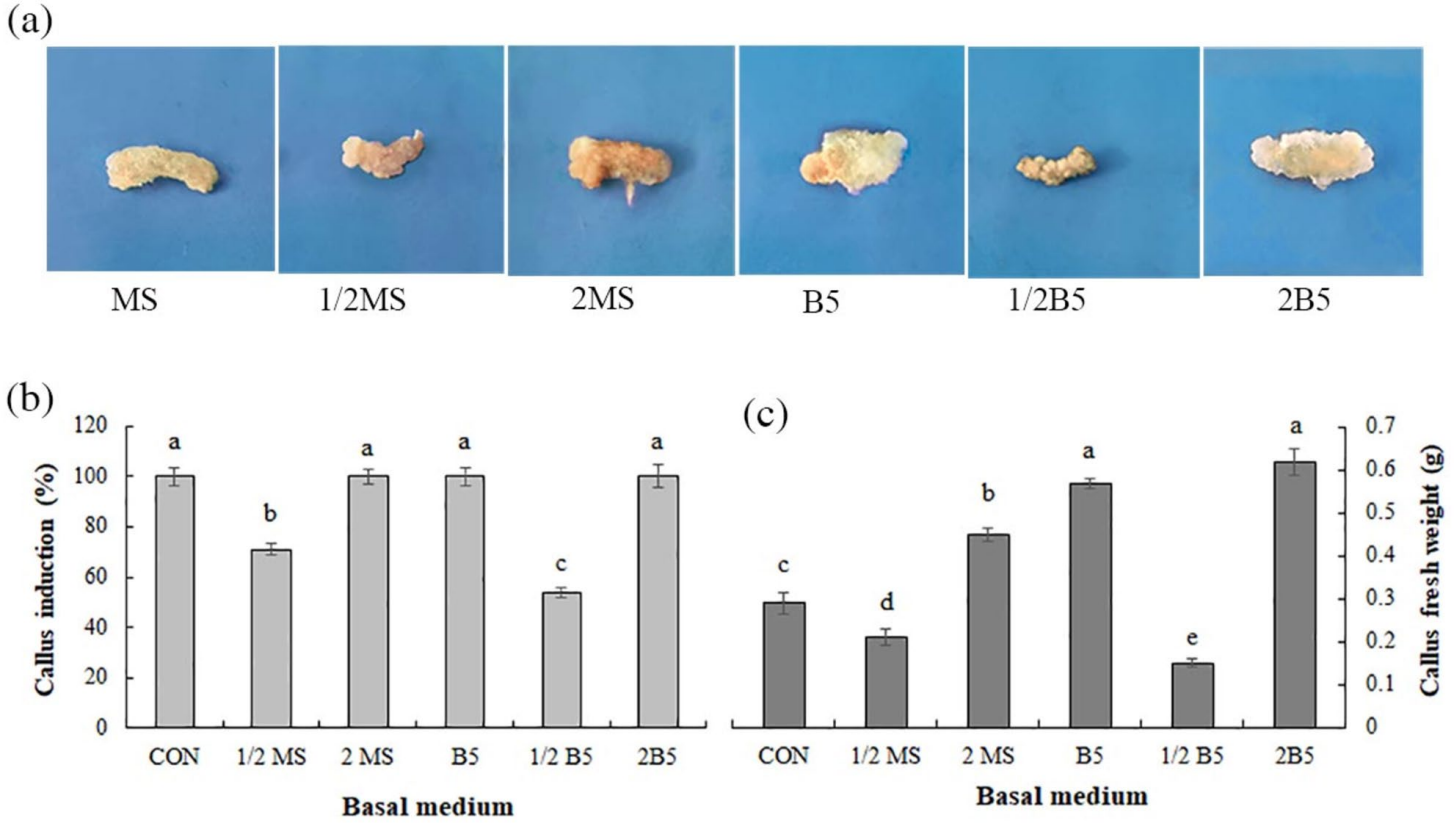
Depiction of *Physalis alkekengi* callus tissues grown under different basal media, (**a**) the impact of basal media on the callus induction (**b**), and callus fresh weight (**c**). Scale bars = 8 cm. Values are means ± SE of five replicates. Different letters indicated significant (p ≤ 0.05) differences. SE; standard error, MS; Murashige and Skoog; B5; Gamborg.

Hypocotyl explants displayed various responses in different basal media supplied to CH (200 mg L^−1^) after 3 weeks (Fig. 3). The Gamborg (B5) and 2B5 media revealed more callus weight as compared to other media, and the 2B5 medium showed the optimum condition for callus induction and growth (0.62 g) (Fig. 3a–c)*.* Calluses induced in the 2B5 medium were friable and cream in color, while in the 2MS and 1/2 MS medium, calluses initiated the rooting and had compact status. In the 1/2 MS and 1/2B5 media, explants displayed the lowest callus induction and trend to get brownish after 2 weeks. The lowest callus fresh weight (0.15 g) and induction (54%) were obtained in the 1/2 B5 medium.

### H_2_O_2_ and NO contents

**Figure 4:**
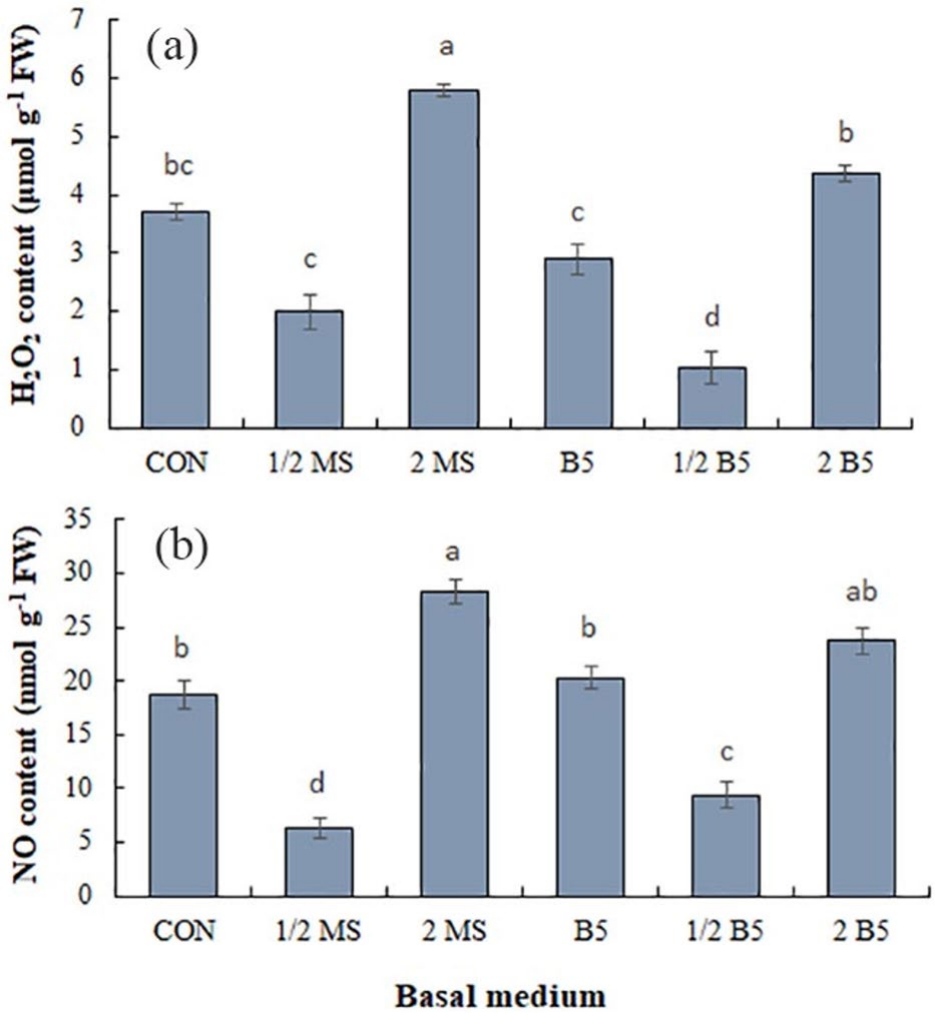
Impact of different basal media on H_2_O_2_ (**b**) and NO (**c**) contents. Values are means ± SE of four replicates. Different letters indicated significant (p ≤ 0.05) differences. SE; standard error.

Signaling molecules of H_2_O_2_ and NO showed considerable alteration under various basal media (Fig. 4). The MS media showed a higher H_2_O_2_ level compared to B5 media (Fig. 4a). The highest H_2_O_2_ content (5.8 µmol g^−1^ FW) was obtained in the 2MS medium with a 56.7.9% rise as compared to control (MS medium, 3.71 µmol g^−1^ FW). The media of B5, 1/2 B5, and 1/2 MS declined the H_2_O_2_ level compared to the control, but in the calluses grown under the 2B5 medium, its content enhanced significantly compared to the B5 medium (*P* ≤ 0.05).

NO content showed various responses under various basal media, As its content was significantly heightened in the 2MS medium with a 1.5-fold rise as compared to the control (Fig. 4b). In the ^1^/_2_MS and ^1^/_2_B5 media, NO level declined about 2.9 and 2-folds, respectively comparing to control.

### Antioxidant enzyme activities

**Figure 5:**
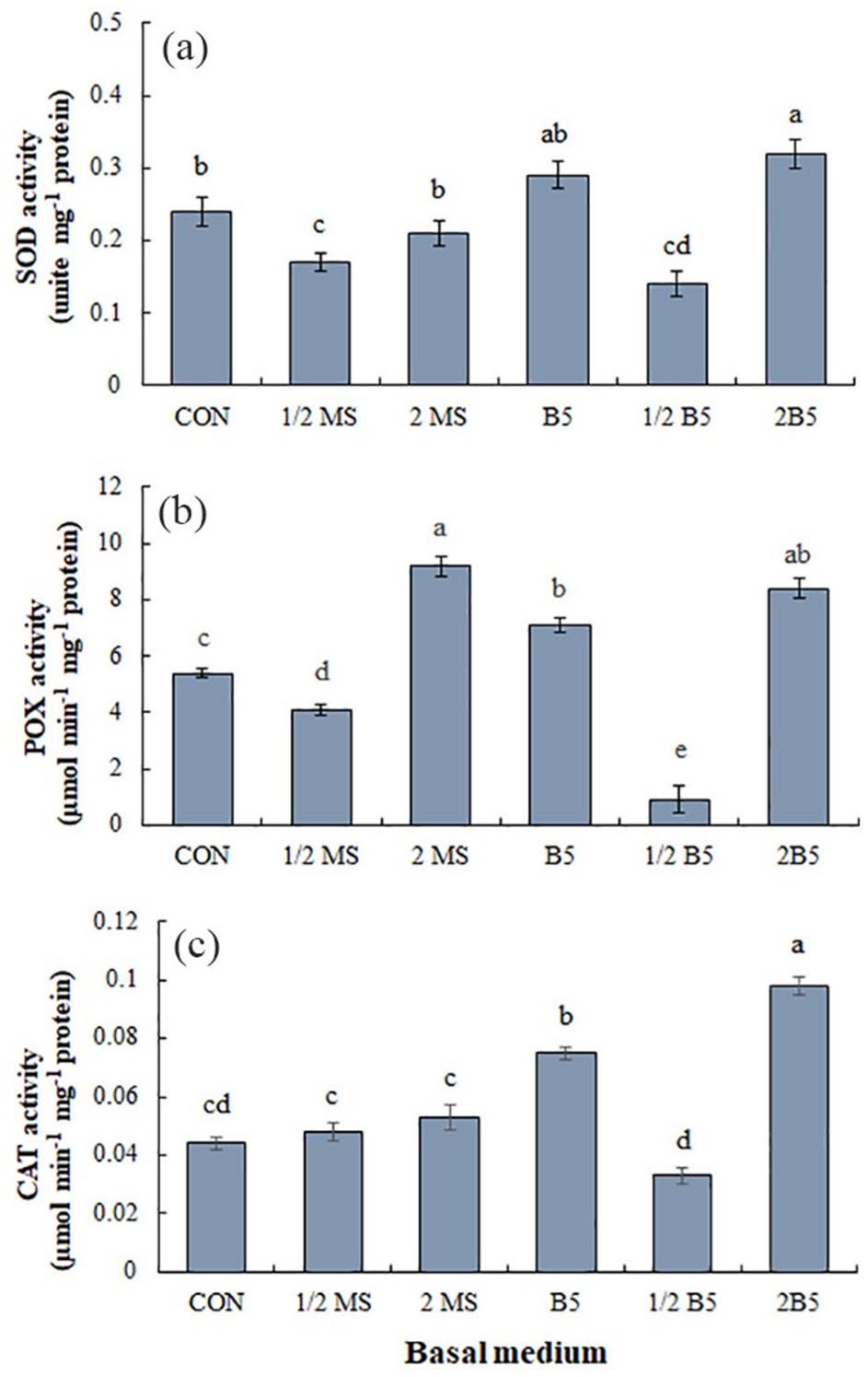
Impact of different basal media on the antioxidant enzyme activities of SOD (**a**), POX (**b**), and CAT (**c**) enzymes. Values are means ± SE of four replicates. Different letters indicated significant (p ≤ 0.05) differences. SE; standard error.

Superoxide dismutase (SOD) activity showed the various responses in the different media. The B5 and 2B5 media increased SOD activity compared to the control (MS medium), and its activity decreased in the 1/2MS and 1/2B5 media (Fig. 5a). The highest SOD activity was obtained in the callus induced in the 2B5 medium with a 33.2% rise compared to control.

The maximum peroxidase (POX) activity was observed under 2MS and 2B5 media with 70.3 and 58.3% rise compared to control, respectively, although its activity was also at a high level under B5 medium (Fig. 5b). In the 1/2MS and 1/2B5 media, the POX activity decreased and was significantly lower than control.

Catalase (CAT) activity increased in the B5 and 2B5 media compared to the control, and the 2B5 medium showed the highest CAT activity with a 2.2-fold rise compared to the control (Fig. 5c). There were not any significant changes in CAT activity in the calluses grown under 1/2B5 medium comparing to control.

### Total phenolic and withanolide contents

Total phenolic content increased significantly in both the 2MS and 2B5 media (P ≤ 0.05), and maximum content (129.44 μg GAE g^−1^ DW) was identified under the 2MS medium (Table 2). In the 1/2 MS and 1/2 B5 media, its content decreased and reached the lowest content in calli grown at 1/2 B5 medium (48.71 μg GAE g^−1^ DW).

**Table 2:** Impact of different basal media in the content of total phenolic and withanolides, DPPH free radical scavenging, and PAL activities of *Physalis alkekengi* callus tissue.

Treatment	Total phenolics (µg GAE g^−1^ DW)	Withanolides (mg g^−1^ DW)	DPPH free radical scavenging activity (%)	PAL activity (mg CA mg^−1^ protein)
MS (Con)	95.32 ± 7.88 b	36.14 ± 2.31 b	68.4 ± 3.72 b	0.52 ± 0.027 b
1/2 MS	62.38 ± 6.35 c	24.71 ± 2.14 c	55.7 ± 2.67 c	0.33 ± 0.024 d
2 MS	129.44 ± 7.18 a	45.72 ± 1.35 a	89.3 ± 4.31 a	0.71 ± 0.032 a
B5	78.62 ± 6.11 bc	43.46 ± 1.95 ab	71.5 ± 3.49 b	0.41 ± 0.039 c
1/2 B5	48.71 ± 4.99 d	27.73 ± 1.04 c	51.4 ± 3.42 c	0.28 ± 0.028 d
2 B5	119.66 ± 8.38 a	49.86 ± 2.45 a	91.3 ± 2.57 a	0.65 ± 0.035 ab

Values are means ± SE of four replicates. Different letters indicated significant (p ≤ 0.05) differences.

Con, control; MS, Murashige and Skoog; B5, Gamborg; SE, standard error; GAE, gallic acid; DW, dry weight; CA, cinnamic acid.

Total withanolide content increased significantly in the calli grown at 2MS and 2B5 media with a 26.5 and 37.9% rise compared to the MS medium (P ≤ 0.05) (Table 2). However, its content reached the lowest content (24.71 mg g^−1^ DW) in the calli grown at 1/2 MS medium with a 46.3% decline compared to the control.

### DPPH free radical scavenging and PAL activities

DPPH (2,2-diphenyl-1-picrylhydrazyl) scavenging activity increased with the accumulation of secondary metabolites in callus tissue. The maximum DPPH activity was observed at the 2B5 medium, with a 33.5% rise compared to the MS medium. The calli grown at 2B5 medium also showed a 30.6% rise compared to the control. However, DPPH activity decreased in the callus tissue grown at 1/2 B5 and ½ MS media with a 24.8 and 18.5% decline compared to the control (Table 2).

PAL (phenylalanine-ammonia lyase) activity increased in the media with the high concentration of nitrogen sources. Similar to the phenolic results, the 2MS medium showed the highest PAL activity, with a 36.5% rise compared to the control. A decrease in the macronutrient level of the medium (^1^/_2_MS and/or ^1^/_2_B5) reduced PAL activity in these media, and the minimum activity was identified in the ^1^/_2_B5 medium.

### Metabolite profile of *P. alkekengi* callus tissue

**Figure 6:**
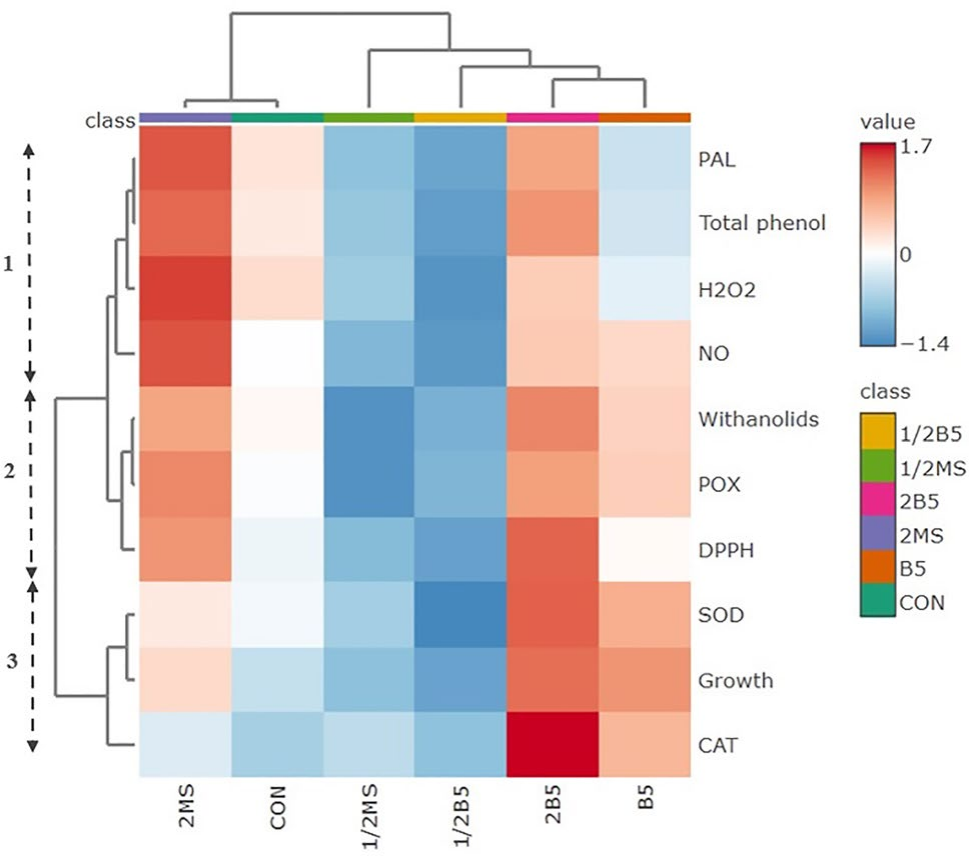
HCA map was employed to cluster antioxidant metabolites and oxidative status under various basal media based on the Pearson correlations coefficient. Colors in matrix boxes represent the direction of correlation: intense red indicates strong positive, and blue indicates strong negative correlations. Three basic clusters are shown from 1 to 3 on the image.

Cluster analysis of different variables required in the callus metabolic responses was shown by the HCA test (Fig. 6). Three clusters have been recognized in which PAL, total phenolics, withaolides, DPPH, NO, H_2_O_2_, and POX are placed in cluster 1 and 2 and CAT, SOD, and growth are placed in cluster 3. It is indicated that the variable distributions may be due to different compositions of basal media. Also, the HCA graph displays a positive relation between signaling molecules, defense metabolites, and growth in the media with high concentrations of nitrogen sources such as 2B5 and 2MS media, although the impact of 2BS medium on the growth induction was higher than 2MS.

**Figure 7:**
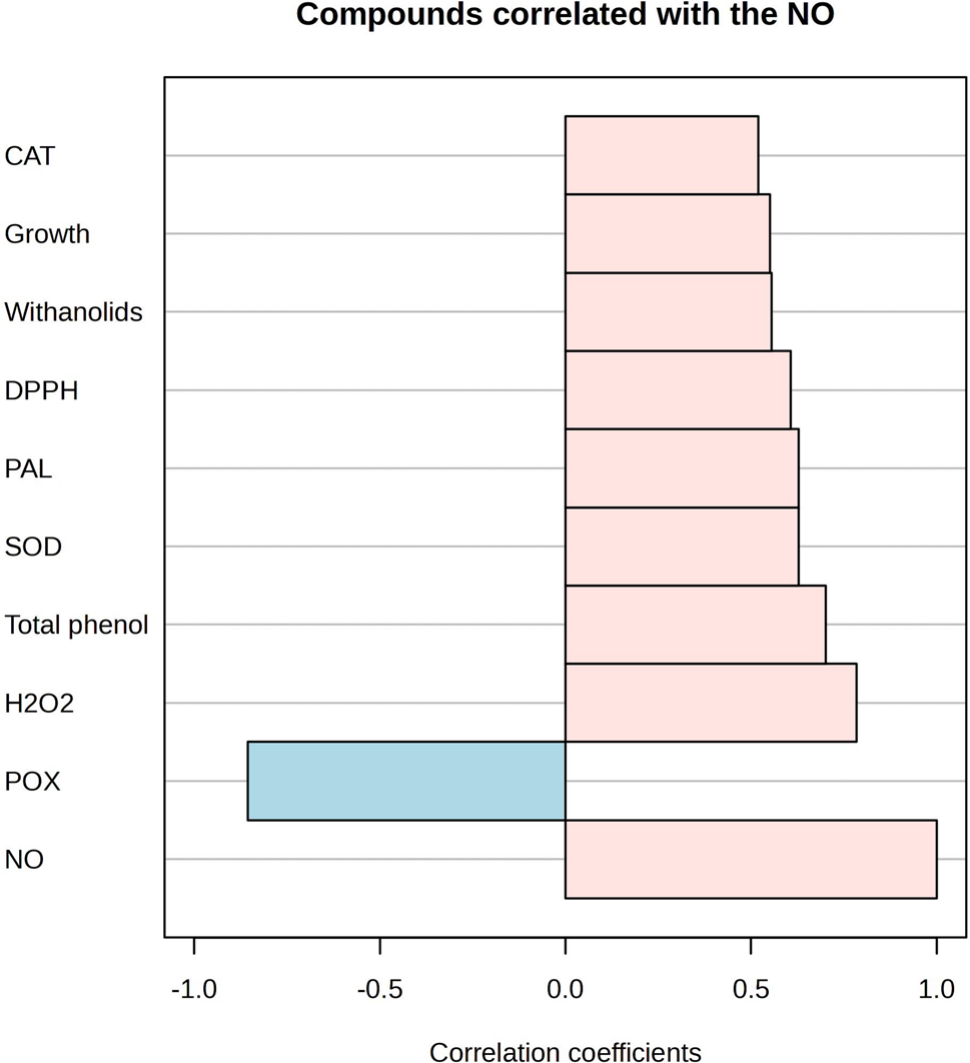
The correlation between NO and other metabolites in the callus grown under various basal media. The cream color indicates a significant positive correlation, and blue has a negative correlation with NO.

A summarized graph in Fig. 7 displays the positive role of NO on the regulation of H_2_O_2_ as a secondary messenger for inducing secondary metabolites, antioxidant enzymes, and growth in *P. alkekengi*. However, NO can also provoke POX activity to increase cell wall lignification and growth reduction.

## Discussion

Plant growth regulators (PGRs) are the crucial factors influencing in vitro callus formation and growth. PGR type and concentration should be optimized in plants based on the explant type and genotype^20^. In this study, optimizing *P. alkekengi* callogenesis showed that medium supplied with picloram decreased callus biomass as compared to medium supplemented with BA (1.5 mgL^−1^) and NAA (0.4 mgL^−1^), and the negative impact of picloram was promoted with the rise of picloram level. In addition, the induced calluses had loose status and got brownish in color at 0.5 and 1 mgL^−1^ concentrations of picloram. There is limited data about callogenesis in *P. alkekengi,* while the impact of picloram has been investigated in some plants. For instance, Kashanchi et al.^15^ reported callus induction of *P. alkekengi* in 1/2 MS medium supplied with 2, 4-D (2 mgL^−1^), and organogenesis was observed during callus growth. Picloram at 1 mgL^−1^ concentration increased markedly days to callus induction and callus diameter in gerbera^21^ and also, in combination with BAP (5 mgL^−1^), induced maximum callus formation in *Simmondsia chinensis*^22^, which may be associated with its impact on cell elongation and division through the responsive gene expressions^23^. However, in *Eupatorium adenophorum*, picloram stimulated electrolyte leakage and malondialdehyde productions and decreased callus biomass at high concentrations^24^. Results of this research showed that picloram is not a suitable growth regulator for callus induction in *P. alkekengi*, reduced the growth rate, and induced calluses to get brownish and loosening, which may be due to the induction of oxidative damage, especially at high concentrations of picloram^24^.

CH (200 mgL^−1^) in the MS medium revealed a positive impact on the callus induction (100%) and biomass (0.46g) in *P. alkekengi*. However, the higher concentrations of CH decreased callus formation and induced organogenesis (Figs. 2 and 3). It has been stated that the medium supplied with CH could induce callus induction and somatic embryo in various plants; however, its concentration should be optimized based on the plant species^13,25^. CH is composed of a mixture of organic compounds, including amino acids, low-molecular-weight proteins, and growth-stimulating factors that promote the growth rate by facilitating cell availability to nitrogen^26^. It has been reported that protein hydrolysates (PHs) participate in various biological processes such as seed germination, shoot regeneration, callus proliferation, somatic embryogenesis, etc.^26,27^. Proteomic analysis displayed that these positive impacts may result from several metabolic convergences associated with phytohormones, redox homeostasis, stress response, primary and secondary metabolic pathways, etc.^28^ In tobacco callus culture, CH showed a functional role as a nitrogen source in callus induction^29^. The findings of this study showed that CH at the concentration of 200 mgL^−1^ can induce a successful callogenesis in *P. alkekengi*, which may be due to the impact on the growth regulators, proteins, and secondary metabolites^30^^.^

**Figure 8:**
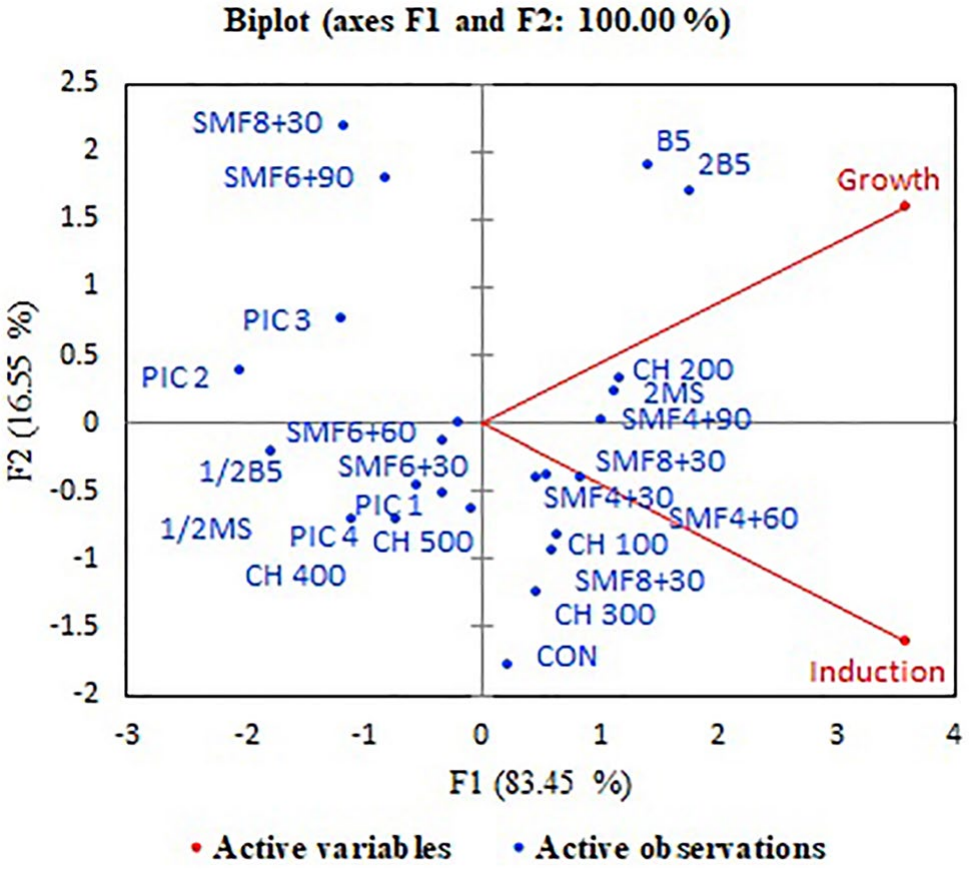
PCA plot of the different factors on the callus induction and growth of *Physalis alkekengi*. Static magnetic field–SMF; Casein hydrolysate–CH; Gambourg–B5; Murashige and Skoog–MS; Picloram–PIC.

In addition, the impact of SMF on the callus biomass was positive, although its potential effect on the callus weight was lower than CH in *P. alkekengi* (Figs. 1 and 2). In the PCA graph, the treatments of CH 200 and SMF4+90 showed a positive impact on the callus growth compared to other treatments (Fig. 8). Both factors of SMF, intensity, and duration, affected the callogenesis, but their impacts were not as a line shape. Explants exposed to 4 mT for 90 min showed a higher growth rate (0.41 g) compared to the 6 and 8 mT. Nasiri et al.^31^ reported that the positive impact of SMF on *Anthemis gilanica* callogenesis was related to the activation of antioxidant enzymes. In *M*. *chamomilla* cell suspension, SMF (4 mT for 1 h) promoted the cell's fresh weight and apigenin production at the late logarithmic phase of growth^16^. SMF increased strawberry growth through the stimulation of membrane permeability and nutrient absorption^19^. The induction of cell division and growth under SMF may be associated with the activation of ROS-mediated signal transduction pathways^32,33^. ROS can regulate the activity of cyclin-dependent kinases (CDKs) and affect the cell cycle progression. Depending on the level of ROS accumulation, CDKs can be inhibited or activated, and following the cell division may promote or delay^34^.

Access to friable and suitable callus may depend on the type and nutrient composition in the culture medium. In this study, the percentage of callogenesis was higher than 70% in various media except for 1/2 B5 medium, which had the lowest callus induction (54%). The B5 and 2B5 media showed an optimum condition for callus growth in *P. alkekengi.* Potassium nitrate (KNO3) is an important macronutrient in the B5 medium, and its requirements vary among species. Unlike MS medium, B5 medium dose not contain ammonium nitrate, have ammonium sulfat, and is increased potassium nitrate level. Findings of this research suggest that KNO3 plays a significant role in *P. alkekengi* callogenesis. Nitrogen is assimilated and applied to produce nucleic acids, proteins, chlorophylls, and coenzymes and can affect cell growth and division^35^. In *Satureja khuzistanica* cell suspension culture*, the* decline in the concentration of KNO3 of B5 medium (1/2B5 and 1/4B5) decreased cell fresh and dry weights, and the optimum cell growth and rosmarinic acid content were obtained in the B5 medium^36^. In this study, increased cell biomass in the 2B5 medium may be due to the impact of KNO3 concentration on the ATP production for oxidation/reduction reactions, which is responsible for cell division and secondary metabolite production^37^. Moreover, the rate of nicotinic acid, pyridoxine, and thiamine vitamins is higher in the B5 medium compared to MS. Vitamins have catalytic functions in enzyme systems and are also involved in various biological processes, including the biosynthesis of amino acids and proteins^38^, carbohydrate metabolism^39^, growth and development^40^ and can act as cofactor in the carboxylase reactions^38^. As in *Phoenix dactylifera*, the rise of thiamine (0.5 mgL^−1^) and biotin (2 mgL^−1^) levels enhanced the callus growth and embryo frequency^41^. On the other hand, the rooting of calluses in this study was revealed in 1/2MS and 2MS media (Fig. 3). Similarly, Arab et al.^42^ reported that the rooting of *Prunus* was promoted in the MS medium with a half amount of potassium nitrate and full ammonium nitrate and also in the medium with reducing of both potassium and ammonium nitrate. Ammonium and potassium play an important role in rooting, possibly as a result of the alteration of osmotic potential and pH in the medium. Ammonium can induce rooting by decreasing the pH and potassium and modifying the osmotic potential^43,44^.

Antioxidant enzyme activities are altered differently in various media. The maximum activity of SOD and CAT were obtained in the 2B5 and B5 media, and POX in the 2MS medium (Fig. 5). Enzymatic antioxidants are accountable for scavenging ROS, cell protection from damage and may affect cell growth by impacting the ROS level^45^. In this study, the maximum level of H_2_O_2_ and NO were identified in the callus grown under the 2MS medium, which may be associated with the higher level of ammonium in this medium. In contrast to the B5 medium, the ratio of NH_4_^+^/NO_3_^−^ is very high in the 2MS medium and can generate ammonium toxicity in some species, which is conducted by interfering with several mechanisms, including disruption in the acid/base balance, reduction in the protein glycosylation, and acidifying the medium^44^. The considerable accumulation of H_2_O_2_ and NO in the calluses grown in the 2MS medium may present that *P. alkekengi* cells are sensitive to the acidified medium, as the POX activity and compact calluses were identified in this medium. It has been stated that POX activity in ionic form can associated with cell wall lignification. Tightening of the cell wall may stimulate the cellular defense against oxidative stress and reduce the cell size and growth^44^, which is in agreement with the result of 2MS on the reduction of growth through compacting callus tissue. As evidenced in Fig. 7, NO stimulates POX activity, which was negatively related to the growth.

H_2_O_2_ and NO are two signaling molecules that act as mediators in the many physiological responses of plants, including cell division, growth and development, programmed cell death, defense responses against stress, etc.^46,47^. NO is produced from several routes in plants through the reduction of nitrite by nitrate reductase (NR), arginine oxidation by nitric oxide synthase (NOS), and/or non-enzymatic pathways^48,49^. Under stress conditions, NO at a suitable level can activate the antioxidant systems to reduce the damage and protect cells from oxidative stress by inducing the gene expressions related to antioxidant enzymes^49^, whereas a high NO level interacts with ROS to produce substances that damage the structure and function of the cells^50^. H_2_O_2_ was found to induce endogenous NO synthesis by impacting the activity of NR and NOS enzymes^51^. H_2_O_2_ and NO can interact with mitogen-activated protein kinases (MAPKs), Ca^2^^+^ ions, growth regulators and modulate the downstream gene expressions and proteins linked to the antioxidant enzymes, cell division, and growth, etc.^52^ In the 2MS medium, the high level of H_2_O_2_ and NO decreased significantly in fresh weight as compared to the 2B5 medium. The nutrient compositions of the 2B5 medium could regulate the level of signaling molecules to induction of growth as the HCA graph displays that the 2B5 medium with a regulating impact on the signaling molecules promotes positive antioxidant enzyme activities, secondary metabolites, and growth (Fig. 6), while the 2MS medium with an excessive accumulation of H_2_O_2_ and NO could trigger POX activity and antioxidant metabolites and shows the low positive effect on the callus growth of *P. alkekengi*.

In addition, the regulation of antioxidant defense metabolism is closely related to the cellular response to oxidative stress. Signaling molecules with an impact on the stress-activated protein kinase and/or MAPKs can regulate the expression level of specific genes associated with secondary metabolism for managing stress response^53^. Phenolic compounds are potent antioxidant agents that can impact cell growth and development. Under stress conditions, phenolic compounds are accumulated in the cells to scavenge excessive ROS. They have various capacities, including antioxidant, anti-inflammatory, anti-microbial, and anti-cancer^54^. Withanolides are the major compounds in *P. alkekengi*, and can act as an antioxidant molecule to scavenge ROSs. In this study, the contents of total phenolic and withanolide, and DPPH scavenging activity increased markedly in the 2MS and 2B5 media, and more activity of PAL was also observed in these media. Both media are rich in nitrogen sources and can act as the stimulator of secondary metabolite production^26^. Nitrogen at a suitable concentration is metabolized in the cell, but at a high level, it can induce oxidative damage and stimulate the biosynthesis of secondary metabolites. In *Scutellaria baicalensis*, Kim et al.^55^ reported a significant enhancement of flavone production under various basal media (MS, B5, and SH (Schenk and Hildebrandt). Results of this research suggest that the impact of various basal media on the enhancement of antioxidant metabolites may be determined by the regulation of androgen NO levels and the activation of enzymes in their biosynthesis pathways.

## Conclusion

In the present study, attempts were made to develop an effective protocol to access friable calluses in *P. alkekengi.* According to the obtained results, picloram was not a suitable hormone for callus induction and growth. SMF at 4 mT for 90 min and CH200 simulated the friable callus growth and induction, although the impact of CH200 on callogenesis was higher than SMF. The type of culture media was an effective factor for callogenesis in *P. alkekengi,* and the 2B5 medium supplied with CH200 was the best medium to access friable callus with high growth. The nutrient composition of the 2B5 medium promoted callus growth by regulating H_2_O_2_ and NO levels, antioxidant enzyme activities, and secondary metabolites. The 2MS medium induced the rooting and POX activity by boosting H_2_O_2_ accumulation. However, the effect of different basal media on the molecular mechanisms underlying callogenesis and secondary metabolites biosynthesis in in vitro* P. alkekengi* is unclear and needs to be investigated in the future.

## Material and methods

### Plant materials

The *P. alkekengi* (voucher number 975) were gathered from Rahimabad, Rudsar, Gilan province of Iran and identified by Dr. Abbas Hadjiakhoondi in Herbarium of Pharmacy School, Tehran University of Medical Sciences Tehran, Iran. Seeds disinfected in the sodium hypochlorite solution (10%, 10 min), rinsed three times in sterile distilled water, immersed in sulfuric acid 40% for 7 min, and afterward rinsed three times in sterile distilled water. The disinfected seeds were placed in the solid ¼ MS medium^56^ under a temperature of 20 ± 2 °C (day/night), relative humidity of 64%, and 16/8 h photoperiod (light/dark) per day with white LED light (45 µmol^−1^ m^−2^ s^−1^). One week after seed germination, seedlings were used as a source of hypocotyl explants.

### Optimizing the callus induction

In order to optimize the callus induction in *P. alkekengi*, the factorial experiment was carried out based on a completely randomized design with 4–5 replications. Five explants per jar (6 cm diameter and 12 cm height) were put in the medium.

### Growth regulators

Based on the preliminary study, the optimum hormonal condition for callus induction of *P. alkekengi* was obtained in the MS medium supplied with NAA (0.4 mgL^−1^) and BAP (1.5 mgL^−1^) (data is publishing). According to the results obtained from initial experiments, the hypocotyl explants (0.4–0.5 cm) placed in the MS medium supplied with NAA (0.4 mgL^−1^) and BAP (1.5 mgL^−1^) as control and different concentrations of picloram (0.25, 0.5, and 1 mgL^−1^) with or without BAP (1.5 mgL^−1^) (Table 1).

### SMF and CH applications

To access the potential impact of SMF and CH, hypocotyl explants were placed in the solid MS medium supplied with picloram (1 mgL^−1^) and exposed to different SMF intensities (0, 4, and 8 mT) for 30, 60, and 90 min and CH (0, 50, 100, 200, 300, 400, and 500 mgL^−1^). The optimum treatment was selected based on the callus induction and fresh weight after 3 weeks. A callus induction rate of 30 replicates per treatment was calculated according to the Zeng et al.^57^ method.

### Different basal media

According to the results obtained from initial experiments and PCA analysis, CH (200 mgL^−1^) showed a greater potential impact on callus growth and was chosen for investigation in various basal media. So, the hypocotyl explants cultured in the different macronutrient compositions of basal media (MS (control), ^1^/_2_MS, 2MS, B5^58^, ^1^/_2_ B5, and 2B5) supplied with the hormonal compositions of NAA (0.4 mgL^−1^) and BAP (1.5 mgL^−1^) and CH (200 mgL^−1^) (Table 3). After 3 weeks of culture, the induced calluses were harvested for some biological and biochemical analyses, with four to five replications for each analysis.

**Table 3:** Macronutrient compositions used in the MS and B5 media at various concentrations.

Macronutrients (mg L^−1^)	MS	^1^/_2_ MS	2MS	B5	^1^/_2_ B5	2B5
NH_4_NO_3_	1650	825	3300	–	–	–
KNO_3_	1900	950	3800	2500	1250	5000
CaCl_2_	332	166	664	150	75	300
MgSO_4_	180.5	90.25	360	250	125	500
KH_2_PO_4_	170	85	340			
(NH_4_)_2_SO_4_	–	–	–	134	67	268
NaH_2_PO_4_	–	–	–	150	75	300

### H_2_O_2_ and NO

The H_2_O_2_ content was assayed through the method explained by Velikova et al.^59^. Fresh callus tissues (0.2 g) were homogenized in 5 mL of cool trichloroacetic acid (TCA, 0.1% (w/v) in an ice bath and certificated at 12,000 × *g* for 10 min, 4 °C. The reaction mixture was 0.5 mL potassium phosphate buffer (pH 7.0, 10 mM), 1 mL KI (1 M), and 0.2 mL extract. The absorbance was read by a UV–vis spectrophotometer (UNICO, 4802, USA) at 390 nm and calculated by the standard curve of H_2_O_2_.

NO content was evaluated using the procedure reported by Kaur et al.^60^ method. Fresh callus (0.5 g) was homogenized in 3 mL of cool buffer phosphate (100 mM, pH 7) and centrifuged at 10,000 rpm for 10 min, 4 °C. The reaction solution was 0.2 mL extract, 1.8 mL buffer phosphate (pH 7), and 0.2 mL Griess reagent (1% sulfanilamide in 5% phosphoric acid solution and 0.1% *N*-1-naphthyl ethylenediamine dihydrochloride) and incubated at 25 °C for 15 min. The absorption was recorded at 540 nm, and NO level was assessed by the standard curve of sodium nitrite.

### Antioxidant enzyme activity

Fresh calluses (200 mg) were homogenized in Tris–HCl (1 M, pH 6.8) at 4 °C and then were centrifuged at 14,000 × g for 25 min. The supernatants were used to measure the enzyme activities. SOD activity was obtained by the procedure described by Giannopolitis and Ries^61^. The reaction mixture was 0.1 mM EDTA, nitroblue tetrazolium (75 μM), 75 μM riboflavin, potassium phosphate buffer (50 mM), 13 mM methionine, and enzyme extract (0.2 ml). The absorbance was read at 560 nm. CAT activity was evaluated using the procedure explained by Aebi^62^. POX activity was evaluated through the procedure of Abeles and Biles^63^.

### Total withanolides, total phenolic, and DPPH activity

Total withanolides content was identified through the procedure reported by Devkar et al.^64^ method. Dried powder of callus (0.2 g) was homogenized in methanol and then centrifuged at 5000 × g for 10 min. The reaction mixture was a 21 mL color reagent (21.5 g ferric chloride hexahydrate in 100 mL orthophosphoric acid), glacial acetic acid (2 mL), and methanolic extract (1 mL). After incubation in an ice bath (5 min), the absorption was determined at 540 nm, and withanolides content was calculated through a standard curve of cholesterol.

Total phenolic content was evaluated via a procedure explained by Singleton and Rossi^65^. Dried powder of callus (0.5 g) was put in 80% (v/v) methanol for 24 h and incubated in an ultrasonic bath for 15 min and then centrifuged at 5000 × g for 10 min. The extract was used for total phenolic and DPPH assays. The 0.3 mL of methanolic extract was added to the 1.5 mL Folin-Ciocalteu reagent and 1.2 mL sodium carbonate (0.7 M). After 30 min, the absorbance was recorded at 760 nm. Total phenolic content was calculated using gallic acid (GAE) as standard.

DPPH scavenging activity was assayed according to the procedure of Rastegaran et al. method^66^, and the absorption was measured at 517 nm. The percentage of inhibition was evaluated by the following equation:$\% {\text{ Inhibition}}\, = \,{\text{AD}} -{ {\text{AS}}/{\text{AD}}}\, \times \,100.$

AS is the absorbance of the sample, and AD is the absorbance of DPPH.

### PAL activity

PAL activity was assayed with the extraction of fresh callus (0.2 g) in Tris–HCl buffer (50 Mm, pH 8.8) supplemented with 15 mM β-mercaptoethanol^67^. The 100 µL enzyme extract was added to the reaction solution of 100 mM Tris–HCl buffer (pH 8.8) and 10 mM phenylalanine and incubated at 37 °C for 1 h. The absorption was recorded at 270 nm, and enzyme activity was evaluated by the standard curve of cinnamic acid.

### Statistical analysis

All of the data were examined by one-way ANOVA followed by Duncan’s test at *P* ≤ 0.05. The experiments were conducted with four or five replications for growth and other analyses. The statistical difference between means was acquired by Duncan’s multiple range test. The principal component analysis (PCA) test for callus induction and growth was conducted by the XLSTAT 2021.2.2 software. Partial least squares discriminate analysis (PLS-DA), and Hierarchical cluster analysis (HCA) were conducted by MetaboAnalyst software on the website (https://www.metaboanalyst.ca/), and all data were compared by the Pearson correlation coefficient.

### Collection of plants or seeds

In the study, the wild plants were gathered which were neither endangered nor at risk of extinction. The collection was conducted towards providing information that will assist to conservation and reproduction of plant.

### Experimental research and field studies on plants

It is confirmed that the handling is conducted in compliance with relevant institution, national and international guidelines and legislation.

## Data Availability

Data will be made available from the corresponding author upon request.
